# Obesity Severity and Cancer Screening in US Adults

**DOI:** 10.1001/jamanetworkopen.2025.32402

**Published:** 2025-09-17

**Authors:** Florina Corpodean, Michael Kachmar, Shengping Yang, Michael W. Cook, Philip R. Schauer, Vance L. Albaugh

**Affiliations:** 1 Metamor Institute, Pennington Biomedical Research Center, Baton Rouge, Louisiana; 2 Department of Surgery, Louisiana State University Health Sciences Center, New Orleans; 3 Our Lady of the Lake Regional Medical Center, Baton Rouge, Louisiana; 4 Department of Biostatistics, Pennington Biomedical Research Center, Baton Rouge, Louisiana; 5 University Medical Center, New Orleans, Louisiana

## Abstract

This cross-sectional study examines the association of severity of adult obesity with rates of US guideline-recommended cancer screening.

## Introduction

Obesity is a cancer risk factor associated with worse prognosis, reduced treatment effectiveness, and higher mortality.^1,2^ The association of obesity with routine cancer screening nationally remains unexplored. Given the disproportionate increase in obesity severity and its known health disparities,^3,4,5,6^ we examined whether worsening obesity severity is associated with lower cancer screening rates.

## Methods

This cross-sectional study used Behavioral Risk Factor Surveillance System data (2012, 2014, 2016, 2018, and 2020 cycles). The Pennington Biomedical Research Center Institutional Review Board approved this study with a waiver of informed consent as data were deidentified and publicly available. The study followed the STROBE reporting guideline.

US adults (aged ≥18 years) without missing body mass index (BMI; calculated as weight in kilograms divided by height in meters squared) or survey data were included. Cancer screening included sigmoidoscopy and colonoscopy, fecal occult blood test (FOBT), Papanicolaou test, mammography, and prostate-specific antigen (PSA) test based on US Preventive Services Task Force recommendations for the corresponding year. Participants were categorized into 5 BMI groups (18.5-29.9 [reference], 30.0-34.9, 35.0-39.9, 40.0-49.9, and ≥50.0). Individuals with BMI less than 18.5 were excluded.

Survey-weighted Poisson regression models adjusted for age, sex, and self-reported race and ethnicity were used to estimate risk ratios (RRs) and 95% CIs. Prevalence estimates are also reported. The Benjamini-Hochberg procedure was applied for multiple comparisons, with *P* < .05 considered significant. All analyses were conducted between January and June 2025 using R, version 2024.12.0 (R Foundation).

## Results

The study sample included 2 057 525 participants (mean [SD] age, 55.5 [17.9] years; 54.9% female and 45.1% male, 8.0% of Black, 7.8% Hispanic, 77.2% White, and 7.0% other race and ethnicity [American Indian or Alaska Native, Asian, multiracial, or other due to insufficient data]) with a mean (SD) BMI of 28.2 (2.5) (18.5-29.9, 69.2%; 30.0-34.9, 18.9%; 35.0-39.9, 7.3%; 40.0-49.9, 3.9%; ≥50.0, 0.7%). Significant differences in cancer screening were observed across BMI categories (Table 1). Prevalence estimates are shown in Table 2. For Papanicolaou testing, BMI 30.0 to 34.9 was associated with higher screening rates (RR, 1.00 [95% CI, 1.00-1.01]) and 50.0 or higher with significantly reduced screening rates (RR, 0.98 [95% CI, 0.97-0.99]). Mammography rates declined with increasing BMI, with prevalence dropping from 76.8% (95% CI, 76.4%-77.2%) for 30.0 to 34.9 to 70.7% (95% CI, 69.0%-72.4%) for 50.0 or higher, but no association was observed after adjustment. In contrast, FOBT increased steadily with BMI, peaking at 50.0 or higher (RR, 1.13 [95% CI, 1.06-1.19]), and PSA testing showed mixed trends (30.0-34.9: RR 1.02 [95% CI, 1.01-1.03]; 40-49.9: RR, 0.94 [95% CI, 0.92-0.97]). For sigmoidoscopy or colonoscopy, increased rates were associated with BMI 30.0 to 34.5 (RR, 1.02 [95% CI, 1.02-1.03]) and 35.0 to 39.9 (RR, 1.03 [95% CI, 1.03-1.04]), but significantly reduced rates were associated with 50.0 or higher (RR, 0.92 [95% CI, 0.89-0.95]).

**Table 1.** Survey-Weighted Poisson Regression of Cancer Screening Test by BMI Category

Screening test and BMI category	RR (95% CI)	*P* value^a^	Adjusted *P* value^b^
Sigmoidoscopy or colonoscopy			
30.0-34.9	1.02 (1.02-1.03)	<.001	<.001
35.0-39.9	1.03 (1.03-1.04)	<.001	<.001
40.0-49.9	1.01 (0.99-1.02)	.20	>.99
≥50.0	0.92 (0.89-0.95)	<.001	<.001
Fecal occult blood test			
30.0-34.9	1.03 (1.02-1.04)	<.001	<.001
35.0-39.9	1.08 (1.06-1.10)	<.001	<.001
40.0-49.9	1.10 (1.08-1.13)	<.001	<.001
≥50.0	1.13 (1.06-1.19)	<.001	.01
Papanicolaou test			
30.0-34.9	1.00 (1.00-1.01)	<.001	.04
35.0-39.9	1.00 (0.99-1.00)	.58	>.99
40.0-49.9	0.99 (0.99-1.00)	.51	>.99
≥50.0	0.98 (0.97-0.99)	<.001	.02
Mammography			
30.0-34.9	1.00 (0.99-1.01)	.16	>.99
35.0-39.9	1.00 (0.99-1.02)	.18	>.99
40.0-49.9	0.99 (0.98-1.00)	.06	1.00
≥50.0	0.97 (0.96-0.98)	<.001	.14
Prostate-specific antigen test			
30.0-34.9	1.02 (1.01-1.03)	<.001	.004
35.0-39.9	0.99 (0.97-1.01)	.28	>.99
40.0-49.9	0.94 (0.92-0.97)	<.001	<.001
≥50.0	0.89 (0.83-0.96)	<.001	.15

Abbreviations: BMI, body mass index (calculated as weight in kilograms divided by height in meters squared); RR, risk ratio.

^a^ Raw *P* values.

^b^ *P* values adjusted using Benjamini-Hochberg procedure for multiple comparisons. Adjusted for age, sex, and race and ethnicity.

**Table 2.** Cancer Screening Prevalence Estimates by BMI Category

Screening test	Estimated prevalence, % (95% CI)				
	BMI 18.5-29.9	BMI 30.0-34.9	BMI 35.0-39.9	BMI 40-49.9	BMI ≥50.0
Sigmoidoscopy or colonoscopy	70.4 (70.2-70.6)	71.2 (70.8-71.5)	71.1 (70.6-71.7)	68.4 (67.6-69.2)	59.3 (57.2-61.4)
Fecal occult blood test	34.1 (33.9-34.6)	34.3 (33.9-34.6)	34.9 (34.4-35.6)	34.9 (34.0-35.7)	33.1 (31.1-35.1)
Papanicolaou test	92.6 (92.4-92.7)	94.8 (94.6-95.0)	94.8 (94.4-95.1)	94.7 (94.3-95.1)	93.4 (92.5-94.4)
Mammography	71.0 (70.8-71.2)	76.8 (76.4-77.2)	75.4 (74.8-76.0)	72.8 (72.0-73.5)	70.7 (69.0-72.4)
Prostate-specific antigen test	57.0 (56.7-57.3)	56.8 (56.3-57.3)	53.9 (53.0-54.8)	48.7 (47.4-50.0)	41.7 (38.3-45.1)

Abbreviation: BMI, body mass index (calculated as weight in kilograms divided by height in meters squared).

## Discussion

This cross-sectional study found that severe obesity was associated with a lower prevalence of some guideline-recommended cancer screenings. While mammography prevalence also declined with increases in BMI, the difference was not significant, which is concerning with rapid rises in severe obesity.^3,4^ In contrast, comparable or slightly higher screening rates were associated with BMI 30.0 to 39.9 vs the reference, possibly due to greater health care engagement with fewer barriers (eg, mobility limitations, inadequate equipment, weight stigma) than observed with severe obesity. The quality of some tests, particularly mammography, may also decline with greater body size due to technical limitations, further discouraging participation.^6^ The association of FOBT with increased BMI possibly reflects the accessibility of home-based testing, though reliance on FOBT alone is concerning without follow-up colonoscopy. Study strengths included a large, nationally representative sample of individuals with high BMI, who are often underrepresented in research. Limitations included self-reported, cross-sectional data and instances in which small effect sizes may not reflect clinical significance. These findings highlight the need for targeted, equity-focused strategies to improve cancer screening access among individuals with obesity.

## Notes

Supplement.
Data Sharing Statement
