# Supplementary material for: Obesity Severity and Cancer Screening in US Adults
Source: JAMA Netw Open. 2025 Sep 17;8(9):e2532402. doi: 10.1001/jamanetworkopen.2025.32402 (PMC12444554; doi:10.1001/jamanetworkopen.2025.32402)
Supplement: Supplement. — Data Sharing Statement [file jamanetwopen-e2532402-s001.pdf]

Data Sharing Statement

Corpodean. Obesity Severity and Cancer Screening in US Adults. *JAMA Netw Open*.
Published September 17, 2025. doi:10.1001/jamanetworkopen.2025.32402

Data
Data available: Yes
Data types: Deidentified participant data
How to access data: Data can be accessed on the Centers for Disease Control website. All data is publicly available.
When available: With publication

Supporting Documents
Document types: Statistical/analytic code
How to access documents: Request for analytic code will be provided with email request to the corresponding author (Vance L. Albaugh, MD, PhD - vance.albaugh@pbrc.edu)
When available: With publication

Additional Information
Who can access the data: Anyone requesting the data given that it is publicly available.
Types of analyses: Any analysis.
Mechanisms of data availability: With a signed data access agreement.
Any additional restrictions: None.
